# Digestive Vulnerability and Exercise Exposure as Correlates of Gastrointestinal Symptoms and Race Withdrawal in Endurance and Ultra-Endurance Athletes

**DOI:** 10.3390/nu18071033

**Published:** 2026-03-25

**Authors:** Benoit Mauvieux, Elizabeth Mahon, Adrian Markov, Aghilas Slamani, Morgane Fresneau, Anthony Berthou, Eglantine Le Chevert, Jamie Pugh, Ben J. Edwards

**Affiliations:** 1 Normandie Université, UNICAEN, UR 74.80 VERTEX, UFR STAPS, 14000 Caen, France; markov1985@gmx.de (A.M.); slamaniaghilas.pro@gmail.com (A.S.); morgane85.fresneau@gmail.com (M.F.); 2 Research Institute for Sport and Exercise Sciences, Liverpool John Moores University, Liverpool L3 5AH, UK; E.Mahon@ljmu.ac.uk (E.M.); J.Pugh@ljmu.ac.uk (J.P.); B.J.Edwards@ljmu.ac.uk (B.J.E.); 3 Santé & Nutrition, Chemin de la Guiblinière, 44000 Nantes, France; anthony.berthou@gmail.com; 4 Laboratoire Mouvement Sport Santé (M2S), Université Rennes 2, UFR STAPS, 35000 Rennes, France; eglantine.le-chevert@univ-rennes2.fr

**Keywords:** exercise-induced gastrointestinal syndrome, digestive vulnerability, ultra-endurance, race withdrawal, endurance nutrition

## Abstract

**Background:** Gastrointestinal (GI) symptoms are common in endurance and ultra-endurance sports and may impair performance or lead to race withdrawal. While nutritional strategies are frequently emphasized, the respective roles of baseline digestive susceptibility and cumulative exercise exposure remain insufficiently characterized. **Methods:** Two complementary cross-sectional questionnaire-based studies were conducted in endurance athletes. Study 1 included 230 ultra-trail runners and examined determinants of systematic GI symptoms during competition using a composite digestive vulnerability (DV) score reflecting susceptibility indicators. Study 2 included 497 endurance and ultra-endurance athletes from multiple disciplines and investigated multivariable correlates of GI symptoms and GI-related race withdrawal, integrating training-related GI symptoms (proxy of digestive vulnerability), habitual competition duration (≥6 h), sport category and specific digestive symptoms. Logistic regression models were adjusted for age and sex. **Results:** In Study 1, the DV score was independently associated with systematic GI symptoms during competition (adjusted OR per point = 1.93, 95% CI 1.33–2.80). In Study 2, athletes reporting GI symptoms during training had markedly higher odds of experiencing GI symptoms during competition (adjusted OR = 3.96, 95% CI 2.67–5.87). Habitual exposure to events lasting ≥6 h was independently associated with increased odds of GI-related race withdrawal (adjusted OR = 2.25, 95% CI 1.35–3.78). GI symptoms during competition represented the strongest proximal correlate of withdrawal (adjusted OR = 7.04, 95% CI 4.00–12.30), indicating a sequential relationship between baseline digestive vulnerability, symptom expression during competition and race termination. After adjustment for digestive vulnerability and exercise exposure, no individual nutritional category remained independently associated with GI outcomes. **Conclusions:** Gastrointestinal symptoms and race withdrawal in endurance athletes were more consistently associated with digestive vulnerability expressed during training and cumulative exercise exposure than with isolated nutritional items. These findings support a vulnerability–exposure framework in which individual digestive susceptibility interacts with prolonged physiological stress during endurance exercise. Identifying athletes with elevated digestive vulnerability during training may represent a practical strategy to improve individualized nutritional preparation and reduce GI-related race interruption.

## 1. Introduction

Trail running and ultra-endurance events have expanded markedly over the past two decades. These competitions are characterized by prolonged exercise durations—frequently exceeding six hours—substantial elevation gain, and sustained metabolic and thermoregulatory strain [1,2]. Despite their growing popularity, dropout rates remain high, and gastrointestinal (GI) symptoms are frequently reported as important contributors to performance impairment and race withdrawal in long-distance events [3,4,5]. Despite increasing scientific attention, the determinants of GI symptoms during prolonged endurance exercise remain incompletely understood, particularly regarding the relative contributions of cumulative physiological exposure, individual digestive susceptibility, and nutritional practices.

Exercise-associated GI symptoms encompass a spectrum of upper and lower gastrointestinal symptoms, including nausea, vomiting, gastroesophageal reflux, abdominal pain, bloating, diarrhoea, and urgency to defecate [6]. Reported prevalence in ultra-endurance settings is high, with substantial proportions of athletes experiencing GI distress during competition and a notable fraction of non-finishers attributing withdrawal to digestive symptoms [5]. Nevertheless, prevalence estimates vary widely (approximately 11–80%) depending on symptom definitions, severity thresholds, timing of assessment, and methodological approaches [7]. While most studies have described the occurrence of GI symptoms, fewer investigations have examined the hierarchical determinants that may contribute to symptom development and race withdrawal under real-world exposure conditions.

From a mechanistic perspective, gastrointestinal disturbances during prolonged exercise are commonly interpreted within the framework of Exercise-Induced Gastrointestinal Syndrome (EIGS) [8]. Redistribution of blood flow toward active skeletal muscle and skin results in marked splanchnic hypoperfusion, with reductions in mesenteric blood flow of up to 70–80% [9,10]. Sustained hypoperfusion may induce epithelial injury, increased intestinal permeability, and inflammatory responses. Beyond circulatory redistribution alone, systematic evidence indicates that exercise per se increases biomarkers of intestinal epithelial injury and permeability, with effects amplified under heat stress conditions, further reinforcing the pathophysiological basis of EIGS [11]. Recent integrative analyses further emphasize the central role of intestinal barrier disruption, endotoxemia, and inflammatory signaling in exercise-induced gastrointestinal stress, while highlighting substantial inter-individual variability in biomarker responses [12]. Reperfusion phenomena, oxidative stress, hyperthermia, environmental heat exposure, and repetitive mechanical impacts—particularly in running-based disciplines—may further exacerbate gastrointestinal strain.

Under these conditions, nutritional factors such as high carbohydrate loads, hyperosmolar solutions, fat- or protein-rich foods, inadequate hydration and ingestion timing may interact with reduced intestinal perfusion. Importantly, these nutritional factors likely interact with an already physiologically stressed gastrointestinal system rather than independently determining symptom occurrence.

In applied settings, GI symptoms are frequently attributed to specific foods or carbohydrate intake rates during competition. While inappropriate nutritional practices can provoke symptoms [6], contemporary reviews of carbohydrate supplementation strategies emphasize that optimal performance nutrition requires individualized approaches that account for gastrointestinal tolerance, exercise intensity, duration and environmental constraints [13]. These observations suggest that fueling strategies operate within a broader physiological context rather than acting as isolated determinants of GI symptoms. Accordingly, evidence supporting a consistent and independent causal role of specific food categories under typical ultra-endurance conditions remains heterogeneous. This discrepancy raises the possibility that nutritional factors may act more as modulators of symptoms in individuals with pre-existing digestive susceptibility rather than as primary causal triggers.

Ultra-endurance exposure is often operationalised as continuous exercise lasting ≥6 h, reflecting cumulative metabolic, thermal, mechanical and circulatory stress rather than an abrupt physiological threshold. In endurance sport research, this duration is frequently used as a pragmatic operational boundary because events exceeding approximately six hours typically involve sustained splanchnic hypoperfusion, progressive metabolic strain and increasing risk of gastrointestinal perturbation.

This variability suggests the existence of an individual digestive vulnerability profile. Digestive vulnerability may be reflected in recurrent GI symptoms during training, heightened digestive sensitivity, or increased symptom reactivity under physiological stress. No single validated instrument formally termed a “Digestive Vulnerability Scale” currently exists. Instead, the construct overlaps with domains assessed in validated questionnaires such as the Gastrointestinal Symptom Rating Scale (GSRS), the Gastrointestinal Symptom Questionnaire (GSQ), and the Visceral Sensitivity Index (VSI). However, these instruments were not specifically designed to characterize digestive susceptibility in ultra-endurance contexts or to predict race withdrawal under prolonged physiological exposure.

Establishing whether recurrent GI distress during training represents a stable marker of baseline digestive vulnerability that predicts competition symptoms and withdrawal is therefore critical. Moving beyond the question of “what was eaten,” understanding how baseline digestive susceptibility interacts with cumulative exposure may provide a more coherent explanatory framework for GI symptoms in endurance and ultra-endurance sport.

The present study does not aim to validate a standardized digestive vulnerability scale. Rather, it tests whether training-expressed gastrointestinal disturbances function as a pragmatic marker of susceptibility under prolonged physiological stress within endurance settings. The aim of the present study was to examine whether digestive vulnerability expressed during training and cumulative exercise exposure are associated with gastrointestinal symptoms during competition and race withdrawal in endurance and ultra-endurance athletes.

### Study Objectives

Building on the vulnerability–exposure framework outlined above, the present investigation aimed to examine the relative contributions of nutritional practices, baseline digestive vulnerability, and cumulative exercise exposure to the occurrence of gastrointestinal symptoms during endurance and ultra-endurance competition.

Specifically, Study 1 investigated determinants of systematic gastrointestinal symptoms during competition in ultra-endurance athletes, with particular attention to the respective roles of fueling practices and baseline digestive vulnerability expressed through recurrent gastrointestinal symptoms during training and symptom reactivity to exercise duration or intensity. Study 2 extended this framework by examining multivariable correlates of gastrointestinal-related race withdrawal across endurance disciplines, integrating digestive vulnerability, habitual exercise exposure (≥6 h), sport category, and specific digestive symptoms.

Digestive vulnerability was conceptualised as a functional susceptibility profile reflected by recurrent or stress-reactive gastrointestinal symptoms rather than as a clinical diagnosis derived from a validated medical scale.

Based on this framework, we hypothesised that gastrointestinal symptoms during competition would be more strongly associated with baseline digestive vulnerability and cumulative exercise exposure than with isolated nutritional items, and that gastrointestinal symptoms occurring during competition would represent the strongest proximal correlate of race withdrawal.

## 2. Materials and Methods

### 2.1. Study Design and Ethical Approval

The present investigation comprised two complementary cross-sectional observational studies conducted using anonymous, non-interventional, structured self-administered online questionnaires targeting endurance and ultra-endurance athletes. No intervention or experimental manipulation was performed in either study.

Both studies were designed and reported in accordance with the STROBE statement for observational research.

The study was conducted in accordance with the Declaration of Helsinki and current European regulations governing personal data protection. The overall research protocol was approved by an independent ethics committee for research (CPP Comité de Protection des Personnes Ouest III, 21-0166, 21.09.61/SIRIPH 2 G 21.01586.000009), and the informed consent of each major participant was obtained. The protocol was conducted according to the Declaration of Helsinki and was regis-tered in Clinical Trials (NCT06297317).

Participation was voluntary and anonymous. Prior to accessing the online questionnaire, participants were presented with a mandatory information page detailing the objectives of the study, eligibility criteria (age ≥ 18 years, ability to read and understand French, and practice of endurance or ultra-endurance sports), the estimated completion time, and information regarding data protection and voluntary participation.

Access to the questionnaire required explicit electronic consent through mandatory confirmation checkboxes indicating that participants:

- Were at least 18 years of age;
- Had read and understood the study information;
- Agreed to participate voluntarily.

Informed consent was therefore obtained electronically before participation.

Data were collected using the Framaforms online platform (Framasoft, France), which complies with the General Data Protection Regulation (GDPR) and ensures secure data hosting within the European Union. No directly identifiable personal data (e.g., name, contact information, or IP address) were collected.

### 2.2. Data Collection Timeframe and Statistical Environment

Participants were recruited using a convenience sampling approach through endurance sport networks, mailing lists, and social media platforms targeting endurance and ultra-endurance athletes. Recruitment messages invited athletes currently engaged in endurance disciplines to participate voluntarily in an anonymous online questionnaire focusing on gastrointestinal symptoms and nutritional practices in endurance contexts.

Data collection took place between 5 January 2023 and 30 April 2023.

The questionnaires were administered using the Framaforms online platform (Framasoft, Lyon, France), which complies with the General Data Protection Regulation (GDPR) and ensures secure data hosting within the European Union.

The questionnaires required approximately 10–12 min to complete for the ultra-trail cohort (Study 1) and 15–20 min for the multisport endurance cohort (Study 2).

Analyses were performed on complete-case datasets, and no imputation of missing data was conducted.

The anonymized datasets from Study 1 and Study 2, including the full questionnaires and accompanying methodological documentation, are publicly available in the Zenodo repository (https://doi.org/10.5281/zenodo.18823509) to promote transparency and reproducibility.

### 2.3. Questionnaire Structure and Variables

The questionnaires used in both studies were developed specifically for the present investigation based on existing literature on exercise-induced gastrointestinal syndrome and sport-related symptom assessment. The instruments were designed to capture gastrointestinal symptoms occurring during training and competition, nutritional practices, and sport exposure characteristics in endurance and ultra-endurance contexts.

The questionnaires do not correspond to previously validated clinical diagnostic scales. Instead, they were constructed as structured epidemiological instruments intended to capture sport-relevant gastrointestinal outcomes under ecological conditions.

Across both studies, the questionnaires collected information in four main domains:

1. **Individual characteristics**—Participants reported demographic information including age and sex.
2. **Sport practice**—Participants provided information regarding their primary discipline, training background, and typical competition formats. In the multisport cohort (Study 2), disciplines were further classified into biomechanical sport groups to explore potential differences in gastrointestinal symptom expression according to movement constraints.
3. **Gastrointestinal symptoms**—Participants reported the occurrence of gastrointestinal symptoms during training and during competition. The questionnaires assessed common upper and lower gastrointestinal manifestations including nausea, vomiting, gastro-oesophageal reflux, abdominal pain, bloating and diarrhoea. Participants were also asked whether gastrointestinal symptoms had previously forced them to abandon a race.
4. **Nutritional practices**—Participants reported food categories and fueling practices used during training or competition, including carbohydrate sources, solid foods and other nutritional strategies typically employed in endurance events.

In addition, participants were asked about the following:

- Self-reported digestive sensitivity in daily life;
- Previous gastrointestinal symptoms during exercise;
- The use of medications taken preventively or during competition to manage digestive symptoms.

The full list of questionnaire items for both studies is available in the publicly accessible dataset deposited in the Zenodo repository, ensuring methodological transparency and reproducibility.

### 2.4. Outcomes and Definitions

The primary gastrointestinal outcomes assessed in the present investigation were the occurrence of gastrointestinal (GI) symptoms during competition and GI-related race withdrawal. GI symptoms during competition were defined as the self-reported occurrence of digestive symptoms experienced during an endurance or ultra-endurance event.

Participants were asked whether they had experienced gastrointestinal disturbances during competition. Reported symptoms included common upper and lower gastrointestinal manifestations frequently described in endurance exercise contexts. Upper gastrointestinal symptoms included nausea, vomiting, and gastro-oesophageal reflux, whereas lower gastrointestinal symptoms included diarrhoea, abdominal pain, and digestive discomfort in the lower abdomen. This classification reflects commonly used distinctions in exercise gastroenterology separating upper and lower GI symptom domains.

The questionnaires did not employ a numerical symptom severity scale. Instead, symptom severity was operationalised pragmatically through the functional impact of symptoms on race continuation. Participants were asked whether gastrointestinal symptoms had previously forced them to abandon a race. Accordingly, gastrointestinal outcomes were interpreted according to two functional levels: the occurrence of GI symptoms during competition and GI-related race withdrawal, the latter reflecting disturbances sufficiently severe to prevent race continuation.

Digestive vulnerability was conceptualised as an individual susceptibility to develop gastrointestinal disturbances under exercise stress. Rather than relying on a validated clinical scale, digestive vulnerability was operationalised through self-reported indicators reflecting digestive sensitivity and recurrent symptom expression in training contexts. The specific operationalisation of this construct differed between Study 1 and Study 2 and is described in the respective study sections below.

### 2.5. Study 1—Ultra-Trail Cohort

#### 2.5.1. Participants

Study 1 targeted ultra-trail runners. Eligible participants were adult athletes (≥18 years) currently practicing ultra-trail running. No upper age limit was applied. As participation in ultra-endurance competitions typically requires a valid medical certificate authorizing sports participation, athletes were assumed to meet standard health eligibility requirements for competitive endurance events.

Participants were recruited through ultra-trail community mailing lists, endurance sport social media groups, and direct email contact with athletes previously involved in ultra-endurance research initiatives.

A total of 230 ultra-trail runners completed the questionnaire during the recruitment period. Analyses were conducted on complete cases. No additional exclusion criteria were applied beyond incomplete questionnaire responses.

Given the exploratory nature of the study, no formal a priori sample size calculation was performed.

#### 2.5.2. Digestive Vulnerability Score

Digestive vulnerability (DV) was operationalised as a composite indicator reflecting self-reported susceptibility to gastrointestinal disturbances during exercise. The DV score was constructed a priori using four binary indicators derived from the questionnaire:

- Gastrointestinal symptoms during training;
- Symptom association with exercise duration;
- Symptom association with exercise intensity;
- Self-reported digestive sensitivity in daily life.

Each item was coded as 0 (absence) or 1 (presence), producing a cumulative score ranging from 0 to 4, with higher values reflecting greater digestive vulnerability. Internal consistency of the four-item composite indicator was assessed using Cronbach’s alpha. The DV score showed a Cronbach’s alpha of 0.66, indicating acceptable internal consistency for an exploratory composite index of digestive susceptibility.

Importantly, systematic GI symptoms during competition were deliberately excluded from the score in order to avoid circularity with the primary outcome.

The DV score does not correspond to a previously validated clinical scale but was designed as a pragmatic composite indicator capturing functional digestive susceptibility in ultra-endurance contexts. The indicators were selected a priori based on their conceptual relevance to gastrointestinal susceptibility during endurance exercise. Specifically, they capture recurrent symptom expression during training, symptom reactivity to exercise stressors (duration and intensity), and general digestive sensitivity in daily life.

These dimensions are consistent with mechanisms described in exercise-induced gastrointestinal syndrome (EIGS) and with domains assessed in validated gastrointestinal symptom questionnaires such as the Gastrointestinal Symptom Rating Scale and related instruments. The DV score should therefore be interpreted as a composite indicator of functional digestive susceptibility rather than a clinically validated diagnostic scale.

For statistical analyses, the DV score was treated as:

- A continuous variable in regression models;
- A categorical variable for graphical illustration of dose–response relationships.

#### 2.5.3. Outcomes

The primary outcome for Study 1 was systematic gastrointestinal symptoms during competition, defined as the recurrent occurrence of at least one gastrointestinal symptom during ultra-endurance races. Reported symptoms included upper and lower gastrointestinal manifestations such as nausea, vomiting, gastroesophageal reflux, abdominal pain, bloating, diarrhea, or urgency to defecate.

A secondary outcome was GI-related race withdrawal, defined as race termination attributed wholly or partially to gastrointestinal symptoms.

In Study 2, gastrointestinal symptoms during competition were defined using the same symptom-based criteria as in Study 1, corresponding to the self-reported occurrence of at least one gastrointestinal symptom during endurance events.

GI-related race withdrawal was defined as race termination reported by the participant as being wholly or partially attributable to gastrointestinal symptoms.

### 2.6. Study 2—Multisport Endurance Cohort

Study 2 included a broader multisport sample of endurance and ultra-endurance athletes. Eligible participants were adult athletes (≥18 years) practicing endurance sports such as running, trail running, cycling, swimming, triathlon, or long-distance trekking. No upper age limit or minimum duration of practice was required. A total of 516 athletes completed the questionnaire during the recruitment period. Participants were excluded if key variables required for the main analyses were missing. Specifically, one participant was excluded due to missing information regarding the primary sport discipline, and eighteen participants were excluded due to missing data for gastrointestinal symptoms during competition. After these exclusions, 497 athletes constituted the analytical sample for models examining GI symptoms during competition, including 326 men (63.2%) and 190 women (36.8%). For analyses examining GI-related race withdrawal, five additional participants were excluded due to missing withdrawal information, resulting in a final analytical sample of 492 athletes.

**Figure 1:**
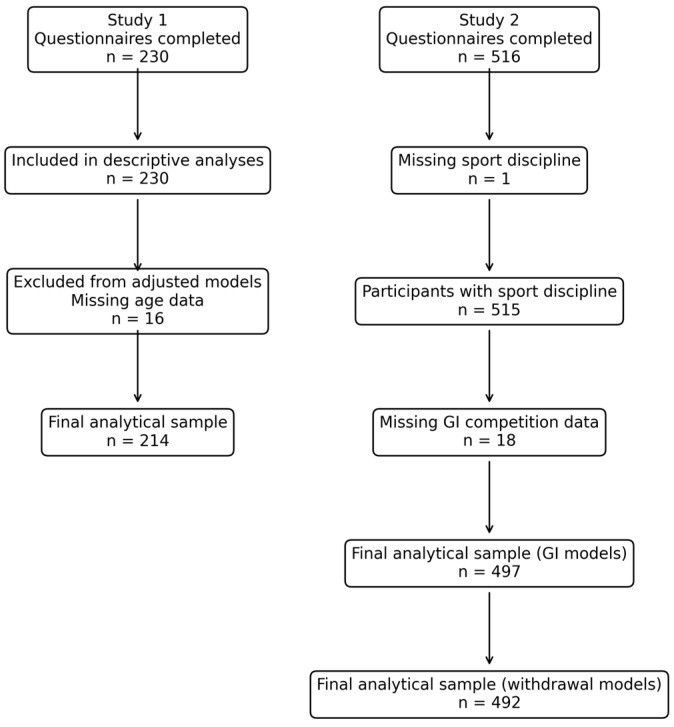
Participant flow diagram for Study 1 and Study 2. Flow diagram illustrating participant recruitment, inclusion, and derivation of the analytical samples for the two cross-sectional observational studies. Study 1 included 230 ultra-trail runners. Study 2 initially included 516 endurance and ultra-endurance athletes, of whom 497 constituted the analytical sample for models examining gastrointestinal (GI) symptoms during competition after exclusion of participants with missing outcome data. Five additional participants had missing information regarding GI-related race withdrawal, resulting in a final sample of 492 athletes for withdrawal analyses.

A flow diagram describing participant inclusion and the derivation of analytical samples is presented in Figure 1.

#### 2.6.1. Digestive Vulnerability Proxy

In Study 2, baseline digestive vulnerability was operationalised using a single indicator: self-reported gastrointestinal symptoms during training (yes/no). This variable was used as a pragmatic proxy for individual susceptibility to gastrointestinal disturbances under exercise stress.

Unlike Study 1, no composite digestive vulnerability score was constructed in this cohort. This simplified operationalisation was intentionally adopted to evaluate whether a single-item indicator of training-expressed gastrointestinal disturbances would replicate the vulnerability–exposure associations observed in the ultra-trail cohort.

#### 2.6.2. Exposure Classification

Habitual competition exposure was categorised according to typical event duration. Events expected to last ≥6 h were classified as ultra-endurance exposure, whereas events lasting <6 h were classified as endurance exposure. This threshold was used as a proxy for cumulative physiological stress, reflecting prolonged metabolic, circulatory, and gastrointestinal strain associated with long-duration exercise. The ≥6 h threshold therefore represents an operational definition of ultra-endurance exposure commonly used in endurance sport research, capturing prolonged cumulative physiological load rather than a strict physiological breakpoint. Classification was based on the typical duration of the athlete’s habitual competition formats (e.g., ultra-distance trail races, 100 km events, Ironman-type triathlon, long-distance cycling events, or multi-hour endurance challenges).

#### 2.6.3. Sport Classification

Sports disciplines were grouped according to biomechanical and postural constraints in order to explore potential differences in gastrointestinal symptom expression. Disciplines were classified into three main categories:

- **Impact sports** (e.g., running and trail running);
- **Mixed sports** (e.g., triathlon and multisport disciplines);
- **Postural or predominantly seated sports** (e.g., cycling).

This classification was used for exploratory analyses examining whether mechanical loading or body posture during exercise may influence gastrointestinal symptom occurrence.

### 2.7. Statistical Analysis

Descriptive statistics are presented as frequencies and percentages for categorical variables. Associations between categorical variables were examined using chi-square tests or Fisher’s exact tests where appropriate.

Multivariable logistic regression models were constructed to examine determinants of gastrointestinal symptoms during competition and determinants of GI-related race withdrawal. Predictor variables were selected a priori based on physiological plausibility and previous literature on exercise-induced gastrointestinal disturbances.

In Study 1, digestive vulnerability was analysed using the composite Digestive Vulnerability (DV) score, whereas in Study 2 baseline vulnerability was operationalised using self-reported gastrointestinal symptoms during training. Training-related GI and the DV score were analysed in separate regression models to avoid conceptual overlap and potential collinearity. The two analyses were conducted on independent datasets corresponding to Study 1 and Study 2.

In Study 1, gastrointestinal symptoms during training constitute one component of the composite Digestive Vulnerability score. In Study 2, this variable was analysed separately as a standalone proxy of digestive vulnerability in order to examine whether training-expressed symptoms alone could reproduce the vulnerability–exposure associations observed in the ultra-trail cohort.

Models were adjusted for age and sex. Results are reported as odds ratios (OR) with 95% confidence intervals (CI). All statistical tests were two-sided, and *p* < 0.05 was considered statistically significant.

For graphical interpretation, the DV score was categorised (0–1, 2, ≥3) to illustrate dose–response relationships with gastrointestinal symptom occurrence.

All analyses were performed using R software (version 4.3.1, R Foundation for Statistical Computing, Vienna, Austria).

Analyses were conducted on complete cases. Missing data were limited and therefore no imputation procedures were applied.

No formal a priori sample size calculation was performed due to the exploratory nature of the study. However, the adequacy of the sample size for multivariable regression analyses was assessed using the events-per-variable (EPV) principle. Recommended thresholds suggest at least ten outcome events per predictor variable to ensure stable model estimates. Given the number of outcome events and the limited number of predictors included in the models, this criterion was satisfied in both Study 1 and Study 2.

Two multivariable models were constructed to address conceptually distinct outcomes: the occurrence of gastrointestinal symptoms during competition and gastrointestinal-related race withdrawal. While both models included common adjustment variables, certain predictors were incorporated only when directly relevant to the outcome under study. In particular, symptom-specific variables were included in the withdrawal model to capture the clinical relevance and severity of gastrointestinal manifestations but were not included in the GI occurrence model in order to avoid conceptual circularity.

## 3. Results

### 3.1. Participant Flow

The derivation of analytical samples for both studies is presented in Figure 1.

### 3.2. Study 1—Ultra-Trail Cohort (N = 230)

#### 3.2.1. Participant Characteristics

A total of 230 ultra-trail runners completed the questionnaire. Mean age was 44.8 ± 10.2 years (range 18–80), and 12.6% were women (29/230). Logistic regression analyses were conducted on complete cases with valid data for age and outcome variables (*N* = 214).

The primary outcome, systematic gastrointestinal (GI) symptoms during competition, was reported by 13.0% of participants (30/230). GI symptoms during training were reported by 12.6% (29/230).

A history of race withdrawal from any cause was reported by 64.3% of participants (148/230). Using a broad definition of digestive withdrawal based on self-reported causes including gastrointestinal symptoms (e.g., nausea, vomiting, diarrhoea), GI-related withdrawal was identified in 24.8% of participants (57/230).

#### 3.2.2. Digestive Vulnerability and GI Symptoms During Competition

A **digestive vulnerability (DV) score** (range 0–4) was constructed a priori from four indicators: GI symptoms during training, symptom association with exercise duration, symptom association with exercise intensity, and self-reported digestive sensitivity. The primary outcome (systematic GI during competition) was excluded from the score to avoid circularity.

**Figure 2:**
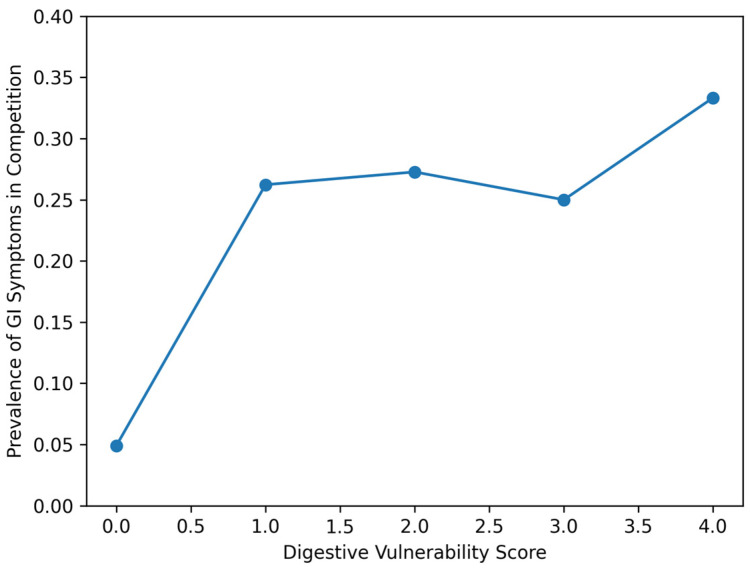
Dose–response relationship between digestive vulnerability and gastrointestinal symptoms during competition in Study 1. Prevalence of gastrointestinal (GI) symptoms during competition according to the Digestive Vulnerability (DV) score in the ultra-trail cohort. The DV score (range 0–4) was constructed from four indicators reflecting baseline digestive susceptibility: GI symptoms during training, symptom association with exercise duration, symptom association with exercise intensity, and self-reported digestive sensitivity. Bar circles represent digestive vulnerability score compared to the prevalence of gastrointestinal symptoms during competition.

The prevalence of systematic GI during competition increased across DV categories (Figure 2):

- **DV 0–1:** 11.3% (23/203);
- **DV 2:** 25.0% (3/12);
- **DV ≥ 3:** 26.7% (4/15).

Although the number of participants in higher DV categories was limited, a progressive increase in systematic GI prevalence was observed across vulnerability levels.

**Figure 3:**
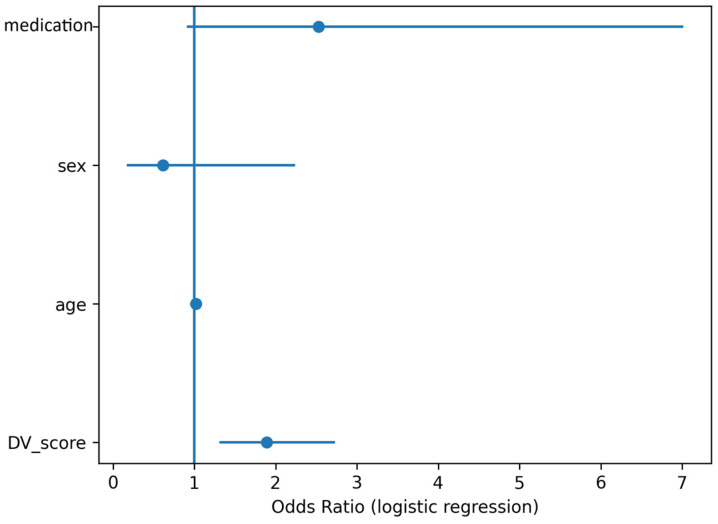
Multivariable predictors of gastrointestinal symptoms during competition in Study 1. Forest plot displaying adjusted odds ratios (OR) and 95% confidence intervals (CI) from the logistic regression model examining determinants of systematic gastrointestinal (GI) symptoms during competition in the ultra-trail cohort. Predictor variables included digestive vulnerability score, age, and sex. The vertical reference line indicates OR = 1.

In minimally adjusted logistic regression models (adjusted for age and sex; complete cases with valid age data, *N* = 214), the DV score analysed as a continuous predictor was independently associated with systematic GI during competition (adjusted OR per +1 point = 1.93, 95% CI 1.33–2.80, *p* = 0.0005) (Figure 3).

#### 3.2.3. Training-Related GI Symptoms and Systematic GI During Competition

Runners reporting GI symptoms during training (*n* = 29) were more likely to report systematic GI during competition: 27.6% (8/29) versus 11.0% (22/201) among runners without training-related GI.

In a minimally adjusted logistic regression model (adjusted for age and sex; *n* = 214), training-related GI symptoms were independently associated with higher odds of systematic GI during competition (adjusted OR = 3.48, 95% CI 1.33–9.14, *p* = 0.011).

To avoid conceptual overlap and potential collinearity, training-related GI and DV score were not included simultaneously in the same regression model but were examined in separate minimally adjusted models.

#### 3.2.4. Nutritional Variables and Systematic GI During Competition

Exploratory analyses examined associations between nutritional items consumed during competition and systematic GI during competition.

In crude analyses, no single food category (sweet products, salty foods, natural foods, industrial products, or carbohydrate beverages) showed a consistent association with systematic GI occurrence.

In multivariable models adjusted for age, sex, and digestive vulnerability (analysed separately to avoid collinearity), no individual nutritional item remained independently associated with systematic GI during competition.

Model comparisons indicated that inclusion of digestive vulnerability substantially improved model fit compared with models including nutritional variables alone, whereas adding nutritional variables to vulnerability-adjusted models produced minimal improvement.

#### 3.2.5. Systematic GI During Competition and GI-Related Withdrawal

Systematic GI during competition was associated with GI-related withdrawal.

**Figure 4:**
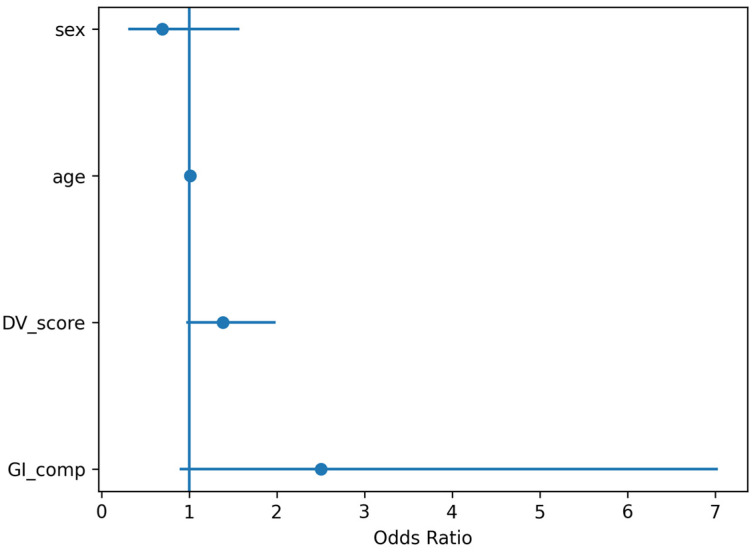
Multivariable predictors of gastrointestinal-related race withdrawal in Study 1. Forest plot showing adjusted odds ratios (OR) and 95% confidence intervals (CI) from the logistic regression model examining determinants of GI-related race withdrawal in the ultra-trail cohort. Predictor variables included gastrointestinal symptoms during competition, age, and sex. The vertical reference line indicates OR = 1.

In crude analyses, systematic GI during competition was associated with higher odds of GI-related withdrawal (OR = 3.19, 95% CI 1.45–7.06). After adjustment for age and sex (*N* = 214), the association remained significant (adjusted OR = 2.96, 95% CI 1.30–6.73, *p* = 0.0097) (Figure 4).

DV score was also associated with GI-related withdrawal in minimally adjusted models (adjusted OR per +1 point = 1.53, 95% CI 1.11–2.10, *p* = 0.0088).

### 3.3. Study 2—Multisport Endurance Cohort

#### 3.3.1. Study Population

A total of 516 athletes completed the online questionnaire. After exclusion of participants with missing data regarding primary sport discipline *(n* = 1) or gastrointestinal (GI) symptoms during competition (*n* = 18), 497 endurance and ultra-endurance athletes constituted the analytical sample for models examining GI symptoms during competition (Figure 1).

Five additional participants had missing data regarding GI-related race withdrawal, resulting in a final analytical sample of 492 athletes for withdrawal analyses.

#### 3.3.2. Sport Distribution and Exposure Duration

The sample was predominantly composed of running-based disciplines. The distribution by primary sport category (*N* = 497) was as follows:

- Trail running: 224 (45.1%).
- Ultra-trail running: 118 (23.7%).
- Road running: 82 (16.5%).
- Triathlon: 44 (8.9%).
- Open-water swimming: 15 (3.0%).
- Cycling: 12 (2.4%).
- Hiking/trekking: 2 (0.4%).

When grouped according to biomechanical constraints, impact-based disciplines represented 85.3% of the sample.

Based on habitual competition formats, 184 participants (37.0%) were classified in the <6 h exposure category, whereas 313 athletes (63.0%) were classified in the ≥6 h exposure category, indicating that a majority of respondents were regularly engaged in ultra-endurance events.

#### 3.3.3. Gastrointestinal Symptoms and Race Withdrawal

GI symptoms during training were reported by 241 of 497 athletes (48.5%).

GI symptoms during competition were reported by 276 of 497 athletes (55.5%).

Overall, 88 of 492 athletes (17.9%) reported at least one GI-related race withdrawal.

Among athletes reporting GI symptoms during competition, 76 of 275 (27.6%) experienced a GI-related withdrawal.

These descriptive results indicate that although GI symptoms were frequent during competition, only a subset of cases resulted in race withdrawal.

#### 3.3.4. Predictors of Gastrointestinal Symptoms During Competition

**Figure 5:**
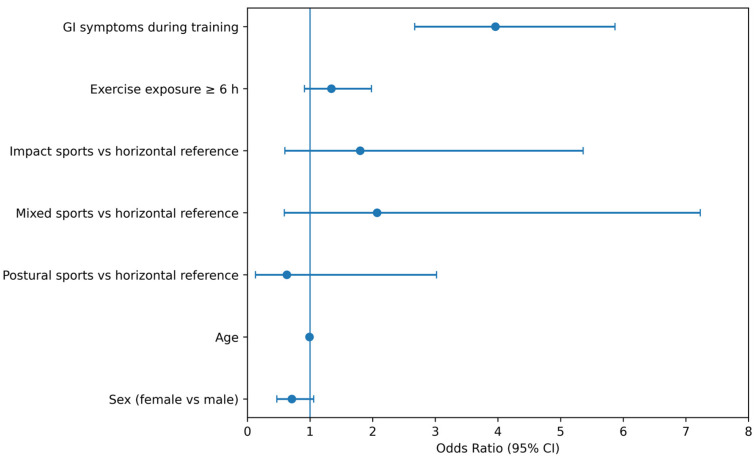
Multivariable predictors of gastrointestinal symptoms during competition in Study 2. Forest plot displaying adjusted odds ratios (OR) and 95% confidence intervals (CI) from the logistic regression model examining determinants of gastrointestinal symptoms during competition in the multisport endurance cohort. Predictor variables included gastrointestinal symptoms during training (proxy for baseline digestive vulnerability), habitual exercise exposure ≥6 h, age, and sex. Horizontal-position sports served as the reference category for sport classification. The vertical reference line indicates OR = 1.

Multivariable logistic regression analysis identified GI symptoms during training as the strongest independent correlate of GI symptoms during competition (Table 1; Figure 5). Athletes reporting GI disturbances during training had markedly higher odds of experiencing GI symptoms during competition (OR = 3.96, 95% CI 2.67–5.87, *p* < 0.001).

Habitual exposure to ultra-endurance events (≥6 h) was associated with higher odds of GI symptoms during competition; however, this association did not reach statistical significance after adjustment (OR = 1.34, 95% CI 0.91–1.98, *p* = 0.13).

Age showed a non-significant trend toward lower odds of gastrointestinal (GI) symptoms with increasing age (OR = 0.99, 95% CI 0.97–1.00, *p* = 0.07). Given the absence of statistical significance, this observation should be interpreted cautiously. Female sex was also not significantly associated with GI occurrence compared with male athletes (OR = 0.71, 95% CI 0.47–1.06, *p* = 0.09).

Sport discipline, grouped according to biomechanical and postural constraints, was not independently associated with GI occurrence after adjustment for exposure duration and GI symptoms during training.

Overall, these results indicate that baseline digestive vulnerability expressed during training represents the primary predictor of GI symptoms during competition, whereas sport type and exposure duration exert more limited independent effects.

#### 3.3.5. Predictors of GI-Related Race Withdrawal

**Figure 6:**
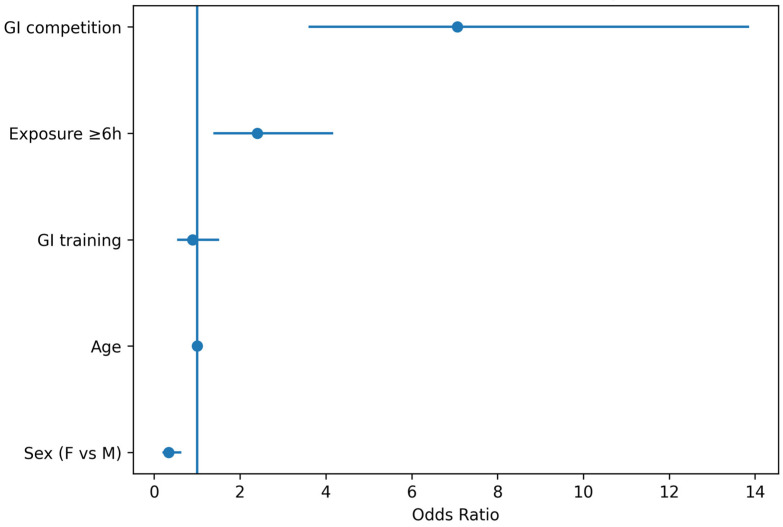
Multivariable predictors of gastrointestinal-related race withdrawal in Study 2. Forest plot displaying adjusted odds ratios (OR) and 95% confidence intervals (CI) from the logistic regression model examining determinants of race withdrawal due to gastrointestinal symptoms in the multisport endurance cohort. The model included baseline digestive vulnerability (GI symptoms during training), exercise exposure ≥6 h, sport discipline category, age, sex, and specific gastrointestinal symptoms reported during competition (vomiting, nausea, diarrhoea, and gastro-oesophageal reflux). The vertical reference line indicates OR = 1.

Multivariable analyses examining determinants of GI-related race withdrawal identified GI symptoms during competition as the strongest predictor of withdrawal (Figure 6). Athletes reporting GI symptoms during competition had substantially higher odds of race withdrawal compared with those without symptoms (adjusted OR = 7.04, 95% CI 4.00–12.30, *p* < 0.001).

Habitual participation in events lasting ≥6 h was independently associated with increased withdrawal risk (adjusted OR = 2.25, 95% CI 1.35–3.78, *p* = 0.002).

When specific digestive symptoms were incorporated into the model, vomiting emerged as a strong predictor of withdrawal (adjusted OR = 4.56, 95% CI 2.40–8.60, *p* < 0.001), and nausea was also independently associated with withdrawal (adjusted OR = 2.34, 95% CI 1.20–4.40, *p* = 0.010). In contrast, diarrhoea and gastro-oesophageal reflux were not independently associated with withdrawal after adjustment.

Taken together, these findings suggest a hierarchical structure in which baseline digestive vulnerability contributes to GI expression during competition, which in turn represents the proximal determinant of race withdrawal, with severe upper GI symptoms acting as key triggers of performance interruption.

## 4. Discussion

### 4.1. Overview of Principal Findings

Across two complementary cohorts, gastrointestinal (GI) symptoms during endurance and ultra-endurance competition were more consistently associated with digestive vulnerability expressed during training and cumulative exercise exposure than with isolated nutritional items.

In Study 1, the digestive vulnerability (DV) score—constructed independently of the primary outcome—was significantly associated with systematic GI during competition (adjusted OR = 1.93 per one-point increase, 95% CI 1.33–2.80). Continuous modelling demonstrated a graded relationship between vulnerability burden and GI expression, supporting a dose–response pattern.

In Study 2, GI symptoms during training independently predicted GI symptoms during competition (adjusted OR = 3.18, 95% CI 2.10–4.82), while habitual exposure ≥6 h independently amplified both GI occurrence (adjusted OR = 1.42) and GI-related withdrawal (adjusted OR = 2.25). GI symptoms during competition represented the strongest proximal correlate of withdrawal (adjusted OR = 7.04).

Taken together, these findings support a hierarchical vulnerability–exposure framework in which digestive susceptibility functions as an upstream determinant, prolonged exposure acts as a cumulative amplifier, and severe symptoms precipitate race termination.

Importantly, no specific nutritional item remained independently associated with GI occurrence after adjustment. This absence of association should not be interpreted as evidence of absence of nutritional effects, but rather as absence of detectable independent effects within the resolution of questionnaire-based assessment.

### 4.2. Duration as Cumulative Exposure

The ≥6 h threshold was used as a pragmatic exposure proxy and does not imply a physiological discontinuity. Splanchnic hypoperfusion and epithelial stress may occur early during exercise [9], but prolonged duration increases cumulative ischemic, thermal, mechanical, and metabolic load.

During sustained endurance exercise, blood flow redistribution toward active skeletal muscles and thermoregulatory skin circulation substantially reduces splanchnic perfusion. Experimental studies have demonstrated that intestinal blood flow may decrease by up to 70–80% during prolonged exercise, potentially leading to epithelial hypoxia and disruption of intestinal barrier integrity [10,14].

These alterations are central components of the exercise-induced gastrointestinal syndrome (EIGS) framework, which describes the cascade linking prolonged exercise, intestinal injury, increased permeability, and systemic inflammatory responses [8,15].

Beyond several hours, sustained blood flow redistribution, hyperthermia, oxidative stress, and repetitive mechanical perturbation increase the probability that subclinical epithelial stress becomes clinically expressed [8,10,16]. Running-related mechanical impacts may further contribute to gastrointestinal disturbance through repetitive visceral displacement and increased intestinal permeability [17].

The persistence of duration-related effects after adjustment for baseline vulnerability suggests that exposure exerts an independent amplifying role rather than merely reflecting athlete self-selection into longer events.

### 4.3. Digestive Vulnerability Expressed During Training

A central and reproducible finding across both studies was the predictive role of training-related GI disturbances. In Study 2, vulnerability was operationalised solely as GI symptoms during training, supporting the interpretation that recurrent symptoms represent a functional marker of susceptibility under physiological stress rather than an isolated race-day phenomenon.

The DV construct was not intended as a psychometric diagnostic scale but as an operational composite reflecting functional susceptibility dimensions relevant to endurance practice. Its purpose was explanatory rather than clinical.

Digestive vulnerability likely reflects multifactorial mechanisms, including exercise-induced gastrointestinal syndrome pathways and inter-individual differences in epithelial resilience, visceral sensitivity, autonomic regulation, and stress reactivity [6,8].

Inter-individual variability in gastrointestinal responses to prolonged exercise has been repeatedly documented and may involve differences in intestinal barrier integrity, autonomic nervous system regulation, inflammatory responses, and microbiome composition [15,18].

Age and sex associations observed in Study 2 further support the contribution of host-related biological variability.

The present design does not allow determination of whether vulnerability reflects intrinsic biological traits, acquired adaptations, or modifiable factors. Prospective studies integrating objective biomarkers (intestinal permeability markers such as I-FABP, zonulin or LBP), microbiome profiling, and multi-omics approaches are needed to clarify whether a biologically distinct digestive vulnerability phenotype can be identified and validated longitudinally.

### 4.4. Symptom Severity and Withdrawal

Although GI symptoms were frequent, only a subset resulted in withdrawal. Incorporation of specific symptoms improved model discrimination (AUC 0.78 to 0.83). Vomiting and nausea emerged as the most proximal determinants of race termination.

This distinction between symptom presence and symptom impact is clinically meaningful. Withdrawal appears to occur when symptom intensity exceeds individual tolerance thresholds, likely involving both physiological disruption and central perception of visceral distress.

Upper gastrointestinal symptoms may also directly compromise fueling and hydration strategies. Severe nausea or vomiting can abruptly interrupt carbohydrate and fluid intake, potentially accelerating energy deficit and dehydration during prolonged exercise [19].

Under these circumstances, digestive symptoms may rapidly translate into performance deterioration and race termination.

### 4.5. Nutrition: Contextualised Rather than Isolated

After adjustment for digestive vulnerability and exercise exposure, no individual nutritional category was independently associated with gastrointestinal outcomes. This result should be interpreted within the limits of the questionnaire-based dietary assessment. Controlled laboratory data demonstrate that even high carbohydrate ingestion rates do not systematically provoke GI distress in trained individuals under standardized conditions [20], supporting the plausibility of substantial inter-individual variability in gastrointestinal tolerance.

However, nutritional assessment lacked detailed quantification of carbohydrate dose (g·h^−1^), osmolality, fiber intake, ingestion timing precision, and habitual dietary patterns. Subtle intake–vulnerability interactions may therefore have remained undetected. Consequently, the absence of independent associations between nutritional categories and gastrointestinal outcomes should be interpreted cautiously and within the resolution limits of questionnaire-based dietary assessment.

Importantly, nutritional behaviour during ultra-endurance competitions is highly dynamic and context-dependent. Athletes frequently alternate between multiple food and beverage sources (energy drinks, gels, bars, solid foods, and fluids provided at aid stations) depending on digestive tolerance, fatigue level, environmental conditions, and psychological preferences during the race.

Under such ecological conditions, precise retrospective quantification of nutrient intake is inherently difficult. Consequently, the absence of independent associations observed here should not be interpreted as evidence of absence of nutritional effects.

Rather, nutritional intake may function as a revealing or amplifying factor in athletes with pre-existing digestive vulnerability. A robust athlete may tolerate diverse fueling strategies, whereas a vulnerable athlete may experience symptoms despite adherence to evidence-based guidelines.

### 4.6. Gut Training

The concept of “gut training,” defined as repeated carbohydrate ingestion during exercise with the aim of enhancing gastrointestinal tolerance and absorptive capacity, has gained increasing attention in sports nutrition [21,22].

Experimental studies suggest that structured carbohydrate exposure during training may increase gastric emptying rates, improve carbohydrate absorption, and enhance exogenous carbohydrate oxidation during prolonged exercise [22].

In the present cohorts, however, self-reported gut-training behaviours were not independently associated with reduced GI burden or lower withdrawal risk after adjustment for age, sex, exposure duration, and digestive vulnerability expressed during training.

These findings should be interpreted cautiously. Questionnaire-based behavioural proxies do not capture the dose, structure, progression, or physiological specificity of gut-training protocols described in experimental studies.

Moreover, gut training may increase tolerance thresholds without eliminating underlying digestive susceptibility. Within the vulnerability–exposure framework proposed here, gut training may shift the boundary at which symptoms become performance-limiting rather than prevent symptom occurrence altogether.

### 4.7. Circadian and Environmental Modulators

Ultra-endurance events frequently extend into the biological night. Gastric motility, enzymatic secretion, and nutrient absorption exhibit circadian modulation, and misalignment between behavioural cycles and endogenous rhythms may impair digestive efficiency [23].

Sleep deprivation and nocturnal exertion may therefore amplify gastrointestinal burden during prolonged competitions.

Environmental heat exposure further exacerbates splanchnic hypoperfusion and epithelial stress, increasing intestinal permeability and systemic inflammatory load [8,24].

These modulators were not systematically quantified in the present study but likely interact with both baseline vulnerability and cumulative exposure.

### 4.8. Strengths and Limitations

This study integrates two complementary cohorts and a hierarchical modelling strategy linking vulnerability, exposure, symptom expression, and withdrawal across endurance disciplines.

Several limitations must nevertheless be acknowledged.

First, the cross-sectional design precludes causal inference. Although the temporal sequence (training → competition → withdrawal) is biologically plausible, the proposed vulnerability–exposure framework should be interpreted as a conceptual model consistent with the observed associations rather than as a demonstrated causal pathway.

Second, all data were self-reported. Voluntary participation through endurance sport networks may introduce self-selection bias, and retrospective symptom reporting may be subject to recall bias.

Third, nutritional assessment lacked fine granularity, limiting interpretation of intake–tolerance interactions.

Fourth, digestive vulnerability was operationalised using questionnaire-based indicators rather than a validated clinical scale.

Fifth, the proportion of female participants in Study 1 was limited (12.6%), potentially constraining sex-specific generalisability. However, this distribution broadly reflects participation patterns commonly observed in ultra-endurance events, particularly ultra-trail races, where female participation typically ranges between approximately 10–20% of starters. Previous studies have nevertheless suggested potential sex-related differences in the prevalence and expression of gastrointestinal symptoms during endurance exercise, which may involve differences in gastrointestinal motility, hormonal regulation, or nutritional tolerance. Study 1 was conducted among athletes engaged in ultra-endurance races, and the sample therefore mirrors the demographic structure of this competitive environment rather than a methodological recruitment bias. In contrast, Study 2 included a broader population of endurance athletes encompassing both events shorter and longer than six hours. Endurance disciplines of shorter duration (e.g., road running, marathon, short-distance triathlon) generally show higher female participation, which likely explains the greater proportion of women observed in this cohort.

Finally, several physiological determinants potentially relevant to digestive vulnerability were not assessed, including habitual dietary patterns, clinical gastrointestinal history, intestinal permeability markers, inflammatory biomarkers, and microbiome composition.

Future studies integrating physiological biomarkers, microbiome profiling, and objective nutritional quantification may help clarify the biological basis of digestive susceptibility during prolonged endurance exercise.

### 4.9. Conceptual and Practical Implications

These findings suggest shifting part of the analytical focus from identifying a single “offending food” toward identifying athletes with training-expressed digestive vulnerability and quantifying cumulative exposure.

This perspective does not negate the importance of fueling strategies. Instead, it situates nutrition within an individualized tolerance framework integrating digestive resilience, exposure load, symptom profile, and environmental context.

From a practical standpoint, endurance athletes reporting recurrent gastrointestinal symptoms during training may represent a subgroup requiring individualized nutritional preparation, progressive fueling adaptation, and careful exposure management during prolonged competitions.

## 5. Conclusions

Across two complementary cohorts, gastrointestinal symptoms and race withdrawal in endurance and ultra-endurance athletes were more consistently associated with digestive vulnerability expressed during training and cumulative exercise exposure than with isolated nutritional items.

Prolonged exercise appears to act as a cumulative amplifier of digestive strain, whereas individual susceptibility determines whether this strain translates into clinically significant symptoms and race withdrawal. Severe upper gastrointestinal manifestations—particularly nausea and vomiting—emerged as the most proximal correlates of race termination.

Together, these findings support a vulnerability–exposure framework in which gastrointestinal outcomes during endurance competitions arise from the interaction between baseline digestive susceptibility and prolonged physiological stress rather than from isolated nutritional factors alone. This perspective may help shift the analytical focus from identifying single nutritional triggers toward understanding how individual susceptibility interacts with cumulative physiological stress during prolonged endurance exercise.

This perspective highlights the importance of individualized digestive profiling and progressive fueling adaptation during training as part of endurance preparation.

Future prospective studies integrating detailed dietary quantification, environmental exposure characterization, and objective physiological biomarkers of intestinal function are needed to further elucidate the biological determinants of digestive vulnerability and identify modifiable risk factors for gastrointestinal complications during prolonged endurance exercise.

## Figures and Tables

**Table 1:** Multivariable logistic regression analysis identifying predictors of gastrointestinal symptoms during competition in Study 2 (multisport endurance cohort). Notes: Odds ratios (OR) were obtained from multivariable logistic regression models examining determinants of gastrointestinal (GI) symptoms during competition. The model included GI symptoms during training (proxy for baseline digestive vulnerability), habitual exercise exposure ≥6 h, age, sex, and sport discipline category. The horizontal sport category served as the reference group for sport classification.

Variable	Odds Ratio (OR)	95% Confidence Interval	*p*-Value
GI symptoms during training	3.96	2.67–5.87	<0.001
Exercise exposure ≥ 6 h	1.34	0.91–1.98	0.13
Age	0.99	0.97–1.00	0.07
Sex (female vs. male)	0.71	0.47–1.06	0.09
Impact sports vs. horizontal reference	1.80	0.60–5.36	0.29
Mixed sports vs. horizontal reference	2.07	0.59–7.23	0.25
Postural sports vs. horizontal reference	0.63	0.13–3.02	0.56

## Data Availability

Anonymised data and additional methodological materials are openly available via the Open Science Framework repository (https://osf.io).
